# LiRA-CD: An open-source dataset for road condition modelling and research

**DOI:** 10.1016/j.dib.2023.109426

**Published:** 2023-07-17

**Authors:** Asmus Skar, Anders M. Vestergaard, Thea Brüsch, Shahrzad Pour, Ekkart Kindler, Tommy Sonne Alstrøm, Uwe Schlotz, Jakob Elsborg Larsen, Matteo Pettinari

**Affiliations:** a Environmental and Resource Engineering, Technical University of Denmark, 2800 Kongens Lyngby, Denmark; b Applied Mathematics and Computer Science, Technical University of Denmark, 2800 Kongens Lyngby, Denmark; c SWECO Denmark, Kokbjerg 5, 6000, Kolding, Denmark; d Danish Road Directorate, Guldalderen 12, 2640 Hedehusene, Denmark

**Keywords:** Live road assessment, Pavement analysis, Road damage detection, Road friction, Road energy consumption, Internet-of-vehicles, Machine-learning, Vehicle dynamics

## Abstract

This data article presents the details of the Live Road Assessment Custom Dataset (LiRA-CD), an open-source dataset for road condition modelling and research. The dataset captures GPS trajectories of a fleet of electric vehicles and their time-series data from 50 different sensors collected on 230 km of highway and urban roads in Copenhagen, Denmark. Additionally, road condition measurements were collected by standard survey vehicles, which serve as high-quality reference data. The in-vehicle measurements were collected onboard with an Internet-of-Things (IoT) device, then periodically transmitted before being saved in a database. Researchers can use the dataset for prediction modelling related to standard road condition parameters such as surface friction and texture, road roughness, road damages, and energy consumption. Furthermore, researchers and pavement engineers can use the dataset as a template for future studies and projects, benchmarking the performance of different algorithms and solving problems of the same type. LiRA-CD is freely available and can be accessed at https://doi.org/10.11583/DTU.c.6659909.

**Specifications Table**

**Table:** 

Subject	Civil and Structural Engineering, Applied Machine Learning, Mathematical Modelling, Data Mining and Statistical Analysis
Specific subject area	Road condition modelling based on in-vehicle sensor data
Type of data	In-vehicle sensor signals from the Renault Zoe electric car (.hdf5 and .txt) and high-resolution reference data from standard surveying vehicles (.csv)
How the data were acquired	In-vehicle sensor data (.hdf5 and .txt) were collected with a AutoPi Telematics Unit (3rd generation) connected to the vehicle's controller area network (CAN) bus. This unit includes a single-board Raspberry Pi computer with added GPS and accelerometer modules. Reference data (.csv) were collected from standard vehicles operated by the Danish Road Directorate (DRD). The surveying included: (i) P79 Profilometer – a van equipped with a beam hosting 25 point lasers that measure longitudinal and transverse profiles; (ii) ARAN9000 – a multi-functional road scanning vehicle that quantifies road defects and distresses using cameras and a Laser Cracking Measurement System (LCMS); and (iii) VIAFRIK – a skid resistance device complying with CEN/TS 15,901–5 standard.
Data format	Raw sensor signals from cars (.hdf5 and .txt) and standard vehicles (.csv)
Description of data collection	In-vehicle sensor data were collected and synchronized automatically with a computer connected to the vehicle's controller area network (CAN) bus and then transmitted to a cloud-based system before being stored in the database
Data source location	City: Copenhagen, Country: Denmark
Data accessibility	Repository name: Live Road Assessment Custom Dataset (LiRA-CD) Data identification number: 10.11583/DTU.c.6659909 Direct URL to data: https://doi.org/10.11583/DTU.c.6659909

## Value of the Data

• The LiRA-CD provides the basis for development of road condition prediction models suitable for wide-area implementation, i.e., models for prediction of surface friction and texture, road roughness, road damages, and energy consumption (see e.g., [5], [6], [7], [8], [9], [10]), and could be useful for road operators and owners, such as road agencies and municipalities.
• The data is suitable for developing new interpretation schemes (e.g., utilizing physical models) and machine-learning algorithms. Researchers can use data to train, validate and test algorithms for estimating road conditions for a variety of road types and conditions.
• The data include high-quality reference measurements from standard surveying vehicles. Hence, it presents a unique opportunity for researchers to link In-vehicle sensor data to standard road condition parameters.
• The reference data may be complemented with images and image datasets (see e.g., [11]). Although this effort requires additional collection and processing of image data, it will increase the quality of training data and enable the utilization of vision-based methods (see e.g., [6]).
• Researchers and pavement engineers can use the dataset as a template for future studies and projects.
• The dataset can be used for benchmarking the performance of different algorithms, solving problems of the same type.

## 2 Objective

This data article aims to provide researchers and pavement engineers with a vehicle dataset that is open-source and suitable for developing large-scale road condition monitoring methods. Specifically, this dataset links in-vehicle sensor data from regular cars to standard road condition parameters utilized by public road agencies (i.e., parameters used as input for planning and managing road networks). Moreover, the article provides visual aids to familiarize readers with the data in the repository and detailed descriptions of the data collected in the LiRA project, including storage formats, file types, and relevant metadata, making the data usable by researchers and engineers worldwide.

## 3 Data Description

**Fig. 1:**
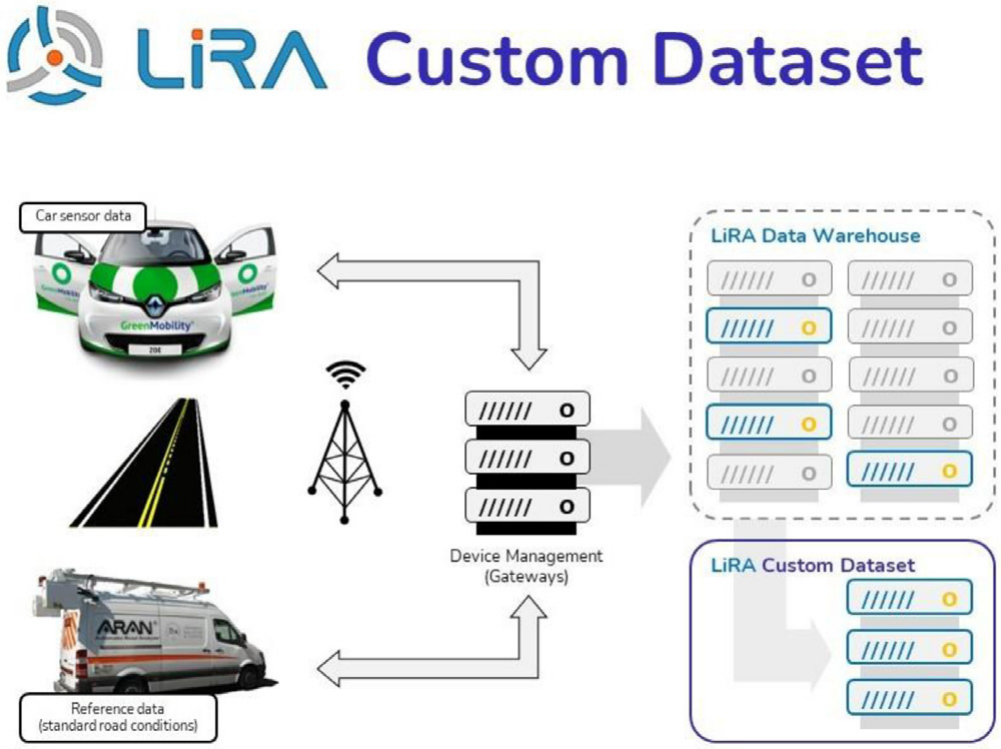
Graphical outline of the data collection and data infrastructure in the LiRA project [1].

The LiRA-CD contains 1796 km of road data from highway and urban roads in the Copenhagen area collected during the LiRA project [1,2]. It includes more than 50 in-vehicle sensor signals from Renault Zoe electric cars operated by GreenMobility (GM) and 92 road condition parameters collected with standard vehicles operated by the Danish Road Directorate (DRD). A Graphical outline of the data collection and data infrastructure is shown in Fig. 1.

The LiRA-CD is a collection of three selected datasets from the LiRA project:

#1 Data subset for road condition modelling - car sensor data. This subset includes the in-vehicle sensor data (.hdf5) collected with an AutoPi Telematics Unit (3rd generation) connected to the vehicle's controller area network (CAN) bus. This unit includes a single-board Raspberry Pi computer with added GPS and accelerometer modules. Moreover, the subset includes a MATLAB® script for accessing and visualizing hdf5-file data.
#2 Data subset for road condition modelling - reference data. This subset includes the reference data, i.e., the standard road condition parameters, collected by vehicles operated by the Danish Road Directorate (DRD). The datasets are divided into subsets, i.e., road elevation data from the P79 Profilometer (P79) (‘..zp…csv’), International Roughness Index (IRI), Mean Profile Depth (MPD) and wheelpath rut depth from the P79 Profilometer (P79) (..iri_mpd_rut…csv’), friction data from the ViaFriction measurement device (VIAFRIK) (‘..fric…csv’), and road condition data from the Automatic Road analyser (ARAN) (‘...aran…csv’).
#3 Data subset for road condition modelling - platoon friction test. This subset includes data from an additional measurement campaign involving a car driving behind the VIAFRIK vehicle. The data are divided into several subsets, i.e., friction data from the VIAFRIK vehicle (‘..fric_custom…csv’) and car sensor data from the AutoPi and the vehicle CAN bus (‘task_7505…txt’).

**Fig. 2:**
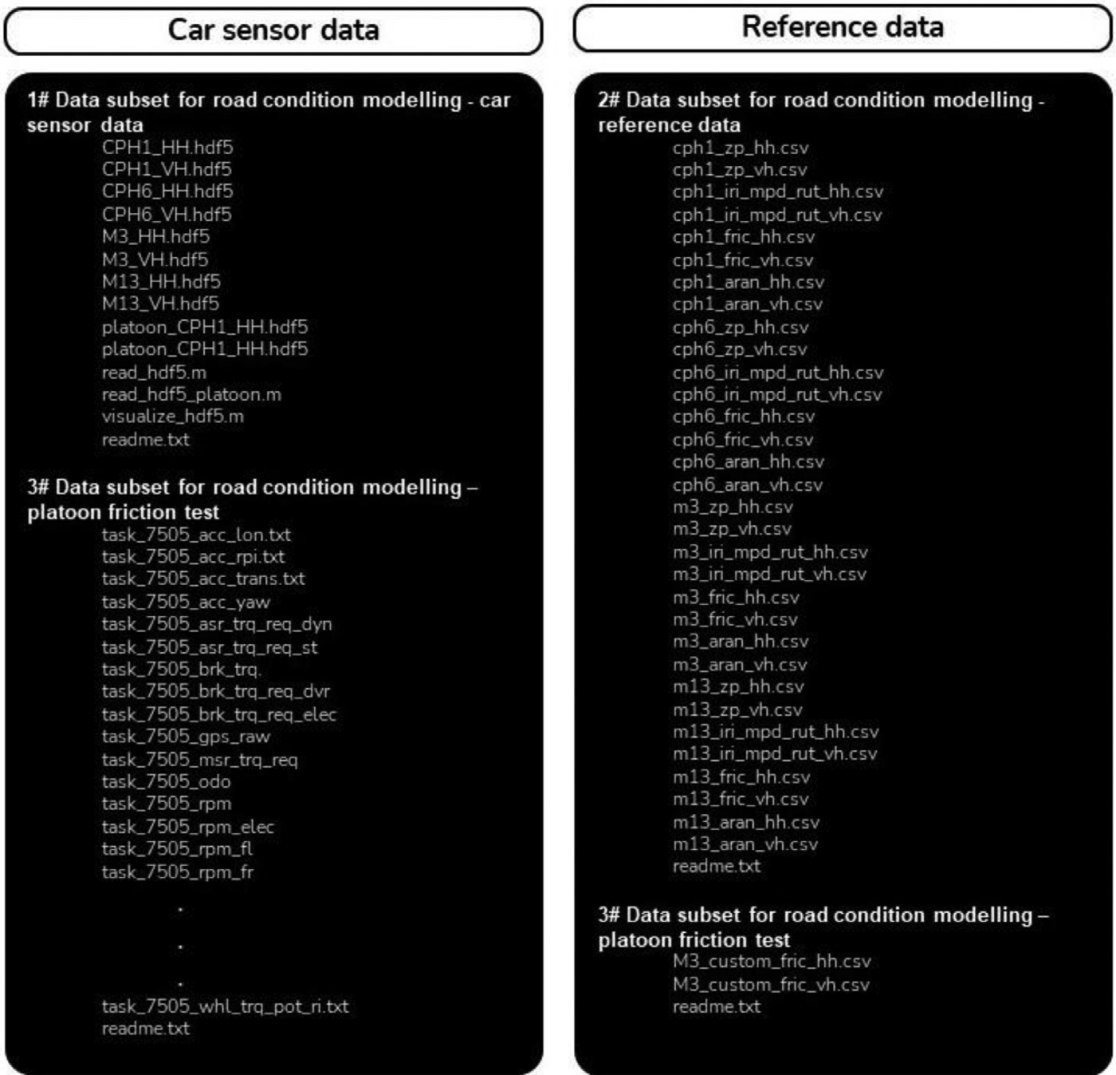
Overview of the LiRA-CD directory and data subsets.

An overview of the LiRA-CD directory and filenames are shown Fig. 2; in the filenames the terms ‘..cph1.. ‘, ‘..cph6.. ‘, ‘..m3.. ‘, and ‘..m13..‘ refers to the road name and, the terms ‘..hh..’ and ‘..vh..’ refers to the right hand side (‘..hh..’) and left hand side (‘..vh..’) of the road (respectively).

**Fig. 3:**
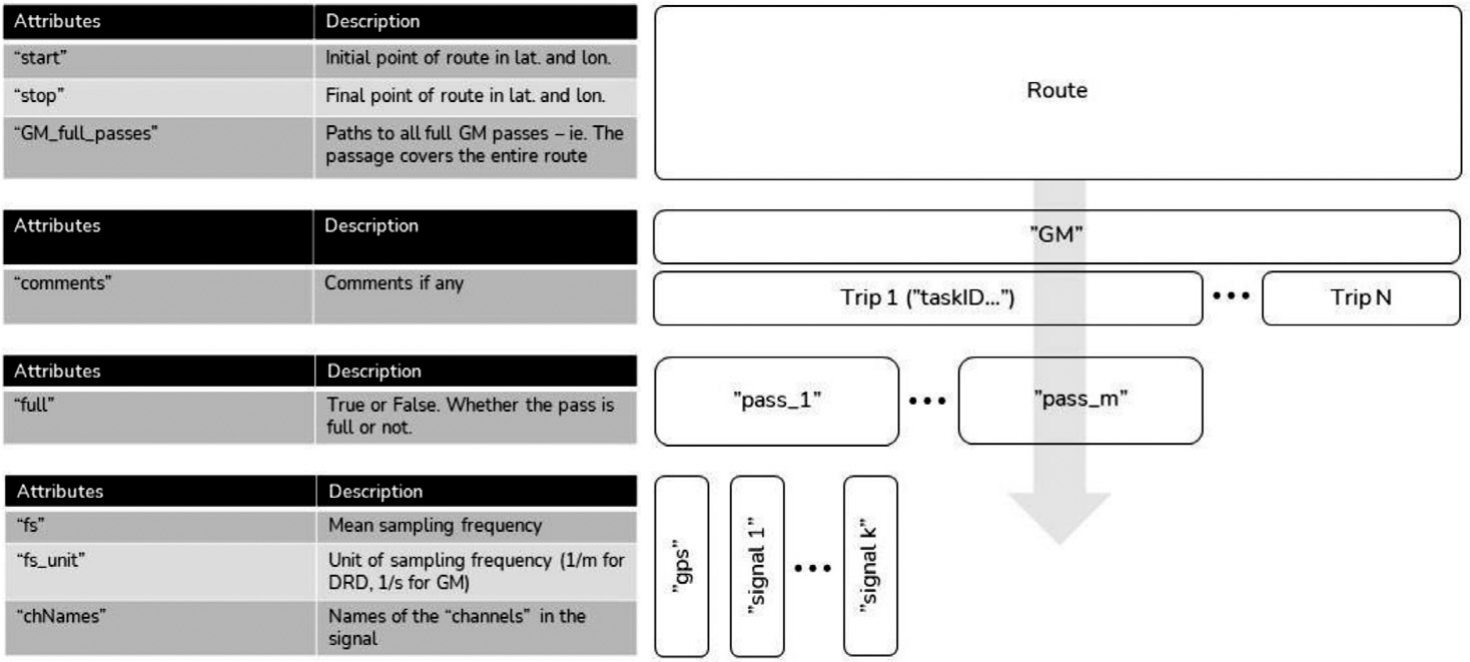
Car data file structure (.hdf5).

The car sensor data is stored in a structured format (.hdf5), an overview of the file structure is depicted in Fig. 3. The Figure shows that the data are structured in five levels; (i) at the top level is route name (i.e., ‘CPH1’, ‘CPH6’, ‘M3’ or ‘M13’), (ii) device/vehicle (in this case on GM for GreenMobility cars), (iii) trip number or task id, (iv) pass number, and (v) GPS and sensor signals. The different signal types and names are described in Table 1.

**Table 1:** Description of car data signal types and names.

Sensor name	Sensor description	Unit
acc.xyz	AutoPi 3D accelerations	g
gps	GPS longitude and latitude	°
obd.strg_pos	Steering Position	°
obd.strg_acc	Steering Acceleration	°/s
obd.strg_ang	Steering Wheel Angle Offset	°
obd.acc_long	Longitudinal Acceleration	m/s^2^
obd.acc_trans	Transversal Acceleration	m/s^2^
obd.acc_yaw	Yaw rate	°/s
obd.brk_trq_req_elec	Electric Brake Wheels Torque Request	Nm
obd.brk_trq_req_dvr	Driver Brake Wheel Torque Request	Nm
obd.whl_trq_est	Estimated Wheel Torque	Nm
obd.trq_eff	Mean Effective Torque	Nm
obd.trq_req	Requested Torque	Nm
obd.whl_trq_pot_ri	Total Potential Resistive Wheels Torque	Nm
obd.brk_trq_elec	Electric Brake Wheels Torque Applied	Nm
obd.asr_trq_req_dyn	ASR Dynamic Torque Request	Nm
obd.asr_trq_req_st	ASR Static Torque Request	Nm
obd.msr_trq_req	MSR Torque Request	Nm
obd.spd_veh	Vehicle Speed	km/h
obd.spd	Speed	km/h
obd.rpm	Engine RPM	rpm
obd.rpm_elec	ElecEngineRPM	Rpm
obd.rpm_fr	Rpm Front Right	rpm
obd.rpm_fl	Rpm Front Left	rpm
obd.rpm_rr	Rpm Rear Right	rpm
obd.rpm_rl	Rpm Rear Left	rpm
obd.whl_prs_rr	Rear right wheel pressure	mbar
obd.whl_prs_rl	Rear left wheel pressure	mbar
obd.whl_prs_fr	Front right wheel pressure	mbar
obd.whl_prs_fl	Front left wheel pressure	mbar
obd.odo	Odometer	km
obd.f_dist	Fine distance	cm
obd.trac_cons	Traction Instant Consumption	kW
obd.cons_avg	Average Consumption	kWh/100km
obd.trip_cons_avg	Average trip B consumpion	kWh/100km
obd.trip_dist	Trip B distance	km
obd.trip_cons	trip B consumption	kWh
obd.trip_spd_avg	Average trip B speed	km/h
obd.ww_f_req	Front Wiping Request	-
obd.ww_f_stat	Front Wiper Stop Position	-
obd.ww_f_stop	Front Wiper Status	-
obd.temp_ext	E xternal Temperature	°C
obd.temp	T emperature	°C
obd.sb_rem_fl	Driver Safety Belt Reminder	-
obd.sb_rem_fr	Front Passenger Safety Belt Reminder	-
obd.sb_stat_rc	Second Row centre Safety Belt State	-
obd.sb_stat_rl	Second Row Left Safety Belt State	-
obd.sb_stat_rr	Second Row Right Safety Belt State	-
obd.time	Local Time	min

The raw car data was translated on-board utilizing a standard On-Board Diagnostics (OBD) protocol [3] and the CanZE application [4]. Processing the data on-board involved converting bits to decimals and then translating decimal data to actual physical units. For some of the car sensors in LiRA-CD, further translation of the sensor signal, s, is required, i.e.:

$$s\;=\;\left(s_{LiRA-CD}-b^{*}\times r^{*}\right)\;-\;b)\;\times r$$

where $s_{LiRA-CD}$ is the sensor signal stored in LiRA-CD, $b^{*}$ and $r^{*}$ are the offset and resolution values (respectively) given in [4], and b and r*,* are the corrected offset and resolution values (respectively) found in the LiRA project. Relevant offset and resolution values are given in Table 2.

**Table 2:** Corrected offset and resolution values utilized to translate car data in the LiRA-CD to physical values.

Sensor name	CanZE [4]		LiRA project	
	b*	r*	*b*	*r*
obd.acc_long	198	1	198	0.05
obd.acc_trans	32,768	1	32,768	0.04
obd.acc_yaw	2047	1	2047	0.1
obd.brk_trq_elec	4096	-1	4098	-1
obd.whl_trq_est	12,800	0.5	12,700	1
obd.trac_cons	80	1	79	1
obd.trip_cons	0	0.1	0	1

The reference data (.csv) is stored in a flat format; each column represents a road condition parameter, GPS information or timestamp, while each row represents a new record in time or space. The different data fields from the reference data, i.e., data collected with the P79 Profilometer (P79), the ViaFriction measurement device (VIAFRIK) and the Automatic Road analyser (ARAN), are described in Table 3, Table. 4, Table. 5, Table 6 (respectively).

**Table 3:** Description of data fields in road elevation data from the P79 vehicle (‘..zp…csv’).

Column header	Description
Distance [m]	Vehicle distance travelled
Laser 1 [mm]	Elevation Laser 1 (transverse position: -1675 mm)
Laser 2 [mm]	Elevation Laser 2 (transverse position: -1475 mm)
Laser 3 [mm]	Elevation Laser 3 (transverse position: -1300 mm)
Laser 4 [mm]	Elevation Laser 4 (transverse position: -1125 mm)
Laser 5 [mm]	Elevation left wheelpath (transverse position: -1025 mm)
Laser 6 [mm]	Elevation Laser 6 (transverse position: -925 mm)
Laser 7 [mm]	Elevation Laser 7 (transverse position: -825 mm)
Laser 8 [mm]	Elevation Laser 8 (transverse position: -725 mm)
Laser 9 [mm]	Elevation Laser 9 (transverse position: -625 mm)
Laser 10 [mm]	Elevation Laser 10 (transverse position: -525 mm)
Laser 11 [mm]	Elevation Laser 11 (transverse position: -425 mm)
Laser 12 [mm]	Elevation Laser 12 (transverse position: -275 mm)
Laser 13 [mm]	Elevation Laser 13 (transverse position: -75 mm)
Laser 14 [mm]	Elevation Laser 14 (transverse position: 125 mm)
Laser 15 [mm]	Elevation Laser 15 (transverse position: 275 mm)
Laser 16 [mm]	Elevation Laser 16 (transverse position: 375 mm
Laser 17 [mm]	Elevation Laser 17 (transverse position: 475 mm)
Laser 18 [mm]	Elevation Laser 18 (transverse position: 575 mm)
Laser 19 [mm]	Elevation Laser 19 (transverse position: 675 mm)
Laser 20 [mm]	Elevation Laser 20 (transverse position: 775 mm)
Laser 21 [mm]	Elevation right wheelpath (transverse position: 875 mm)
Laser 22 [mm]	Elevation Laser 22 (transverse position: 975 mm)
Laser 23 [mm]	Elevation Laser 23 (transverse position: 1150 mm)
Laser 24 [mm]	Elevation Laser 24 (transverse position: 1325 mm)
Laser 25 [mm]	Elevation Laser 25 (transverse position: 1525 mm)
Lat	Latitude
Lon	Longitude
Højde	Elevation
GeoHøjde	Reference height
Alt	Altitude (‘Højde’ + ‘GeoHøjde’)
Bearing	Calculated vehicle heading (bearing angle)

**Table 4:** Description of data fields of International Roughness Index (IRI), Mean Profile Depth (MPD) and wheelpath rut depth from the P79 vehicle (..iri_mpd_rut…csv’).

Column header	Description
Distance [m]	Vehicle distance travelled
IRI (5) [m/km]	Left wheelpath International Roughness Index (IRI)
IRI (21) [m/km]	Right wheelpath International Roughness Index (IRI)
MPD 1	Left wheelpath Mean Profile Depth (MPD)
MPD 2	Right wheelpath Mean Profile Depth (MPD)
Venstre sporkøring [mm]	Left wheelpath rut depth
Højre sporkøring [mm]	Right wheelpath rut depth

**Table 5:** Description of data fields of friction data from the VIAFRIK vehicle (‘..fric…csv’).

Column header	Description
Tid [ms]	Time in milliseconds
µ_V [-]	Frictioncoefficient left wheelpath
µ_H [-]	Frictioncoefficient right wheelpath
v_MW_V [km/t]	Speed left wheel
v_MW_H [km/t]	Speed right wheel
Slip_V [%]	Sliprate left wheel
Slip_H [%]	Sliprate right wheel
v_TW [km/t]	Speed towing wheel
TotalDist [m]	Accumulated distance travelled
Lat	Latitude
Lon	Longitude
F_vertikal_V [N]	Vertical force on left wheel
F_vertikal_H [N]	Vertical force on right wheel
F_friksjon_V [N]	Frictional force on left wheel
F_friksjon_H [N]	Frictional force on right wheel
Bearing	Calculated vehicle heading (bearing angle)

**Table 6:** Description of data fields of road condition data from the ARAN vehicle (‘...aran…csv’).

Column header	Description
L_Route_ID	Unique test identifiction number automatically reported by the ARAN vehicle
DCSTimeStamp	Date and start time of test (number format)
BeginChainage	Chainage at the beginning of measurement window
EndChainage	Chainage at the end of measurement window
Venstre IRI (m/km)	Average Left wheelpath International Roughness Index (IRI)
Højre IRI (m/km)	Average Right wheelpath International Roughness Index (IRI)
Rivninger MeanRI (cm³/m²)	Average Ravelling Index (RI) in cm3/m2
Rivninger MeanExistingRI (cm³/m²)	Average Ravelling Index (RI) in cm3/m2
Rivninger MeanRPI (cm³/m²)	Average Road porosity Index (RPI) in cm3/m2
Rivninger MeanAVC (cm³/m²)	Average Air Void content (AVC) in cm3/m2
venstre wheelpath texture mpd (mm)	average left wheelpath mean profile depth (mpd) from lcms lasers
centre texture mpd (mm)	average road centre mean profile depth (mpd) from lcms lasers
Højre Wheelpath Texture MPD (mm)	Average right wheelpath Mean Profile Depth (MPD) from LCMS lasers
Left Texture Lasers MPD (mm)	Average left wheelpath Mean Profile Depth (MPD) from texture lasers in front of the vehicle
Right Texture Lasers MPD (mm)	Average right wheelpath Mean Profile Depth (MPD) from texture lasers in front of the vehicle (MPD)
Krakeleringer Små (m²)	Sum of low severity crocodile crakcs (0.5 < crack density < 0.8). The area is calculated based on a box surrounding the detected area.
Krakeleringer Middelstore (m²)	Sum of medium severity crocodille crakcs (0.8 < crack density < 1.5) . The area is calculated based on a box surrounding the detected area.
Krakeleringer Store (m²)	Sum of high severity crocodille crakcs (1.5 < crack density < 100). The area is calculated based on a box surrounding the detected area.
Krakeleringer AVG Width (mm)	Average width of crocodile cracks within section
Krakeleringer MIN Width (mm)	Minimum width of crocodile cracks within section
Krakeleringer MAX Width (mm)	Maximum width of crocodile cracks within section
Krakeleringer MIN Depth (mm)	Minimum depth of crocodile cracks within section
Krakeleringer MAX Depth (mm)	Maximum depth of crocodile cracks within section
Revner På Langs Små (m)	Sum of low severity longitudinal cracks (1 mm < crack < 5 mm) within section
Revner På Langs Middelstore (m)	Sum of medium severity longitudinal cracks (5 mm < crack < 30 mm) within section
Revner På Langs Store (m)	Sum of high severity longitudinal cracks (30 mm < crack < 50 mm) within section
Revner På Langs Sealed (m)	Sum of sealed longitudinal cracks within section (detection using smoothness and pixilation differences between the sealed crack and the surrounding area)
Revner På Langs AVG Width (mm)	Average width of longitudinal cracks within section
Revner På Langs MIN Width (mm)	Minimum width of longitudinal cracks within section
Revner På Langs MAX Width (mm)	Maximum width of longitudinal cracks within section
Revner På Langs MIN Depth (mm)	Minimum depth of longitudinal cracks within section
Revner På Langs MAX Depth (mm)	Maximum depth of longitudinal cracks within section
Transverse Low (m)	Sum of low severity transverse cracks (1 mm < crack < 5 mm) within section
Transverse Medium (m)	Sum of medium severity transverse cracks (5 mm < crack < 30 mm) within section
Transverse High (m)	Sum of high severity transverse cracks (30 mm < crack < 50 mm) within section
Transverse Sealed (m)	Sum of sealed transverse cracks within section (detection using smoothness and pixilation differences between the sealed crack and the surrounding area)
Transverse AVG Width (mm)	Average width of transverse cracks within section
Transverse MIN Width (mm)	Minimum width of transverse cracks within section
Transverse MAX Width (mm)	Maximum width of transverse cracks within section
Transverse MIN Depth (mm)	Minimum depth of transverse cracks within section
Transverse MAX Depth (mm)	Maximum depth of transverse cracks within section
Slaghuller Max Depth Low (mm)	Maximum depth of low severity potholes within section
Slaghuller Max Depth Medium (mm)	Maximum depth medium severity potholes within section
Slaghuller Max Depth High (mm)	Maximum depth high severity potholes within section
Slaghuller Max Depth Delamination (mm)	Maximum depth of delamination within section
Slaghuller Area Affected Low (cm²)	Sum of low severity potholes in cm2 (Average depth between 10 mm and 25 mm)
Slaghuller Area Affected Medium (cm²)	Sum of medium severity potholes in cm2 (Average depth between 25 mm and 50 mm)
Slaghuller Area Affected High (cm²)	Sum of high severity potholes in cm2 (Average depth higher than 50 mm)
Slaghuller Area Affected Delamination (cm²)	Sum of delamination in cm2 (Average depth between 0 mm and 10 mm)
Middelstore Bleeding Left Wheel Path (%)	Percentage of area with light bleeding left wheelpath (1.50 < bleeding index < 1.75). The detection utilize smoothness and pixilation differences between the area with bleeding and the surrounding area.
Middelstore Bleeding Right Wheel Path (%)	Percentage of area with light bleeding right wheelpath (1.50 < bleeding index < 1.75). Detection using smoothness and pixilation differences between the area with bleeding and the surrounding area.
Store Bleeding Left Wheel Path (%)	Percentage of area with medium bleeding left wheelpath (1.75 < bleeding index < 2.00). The detection utilize smoothness and pixilation differences between the area with bleeding and the surrounding area.
Store Bleeding Right Wheel Path (%)	Percentage of area with medium bleeding right wheelpath (1.75 < bleeding index < 2.00). The detection utilize smoothness and pixilation differences between the area with bleeding and the surrounding area.
Meget Store Bleeding Left Wheel Path (%)	Percentage of area with severe bleeding left wheelpath (bleeding index > 2.00). The detection utilize smoothness and pixilation differences between the area with bleeding and the surrounding area.
Meget Store Bleeding Right Wheel Path (%)	Percentage of area with severe bleeding right wheelpath (bleeding index > 2.00). The detection utilize smoothness and pixilation differences between the area with bleeding and the surrounding area.
LRUT Straight Edge (mm)	Average rut depth left wheelpath, calculated using the StraightEdge method
RRUT Straight Edge (mm)	Average rut depth right wheelpath, calculated using the StraightEdge method
LRUT Wire (mm)	Average rut depth left wheelpath, calculated using the WIRE method
RRUT Wire (mm)	Average rut depth right wheelpath, calculated using the WIRE method
Venstre Exterior Single Stone (cm²)	Sum of area with single stone (loss) left edge
Venstre Exterior Multi Stone (cm²)	Sum of area with multi stone (loss) left edge
Venstre Wheel Path Single Stone (cm²)	Sum of area with single stone (loss) left wheelpath
Venstre Wheel Path Multi Stone (cm²)	Sum of area with multi stone (loss) left wheelpath
Centre Single Stone (cm²)	Sum of area with single stone (loss) central zone
Centre Multi Stone (cm²)	Sum of area with multi stone (loss) central zone (see road zone definition)
Højre Wheel Path Single Stone (cm²)	Sum of area with single stone (loss) right wheelpath (see road zone definition)
Højre Wheel Path Multi Stone (cm²)	Sum of area with multi stone (loss) right wheelpath
Højre Exterior Single Stone (cm²)	Sum of area with single stone (loss) right edge (see road zone definition)
Højre Exterior Multi Stone (cm²)	Sum of area with multi stone (loss) right edge (see road zone definition)
Latitude From (deg)	Latitude at the start of the measurement window
Latitude To (deg)	Latitude at the end of the measurement window
Longitude From (deg)	Longitude at the start of the measurement window
Longitude To (deg)	Longitude at the end of the measurement window
Heading (deg)	ARAN vehicle heading (bearing angle)
Elevation (m)	Altitude
Lat	Average latitude of the measurement window
Lon	Average longitude of the measurement window
Alt	Altitude
Heading	ARAN vehicle heading (bearing angle)
Bearing	Calculated vehicle heading (bearing angle)

The raw data has not been aligned or structured, hence several pre-processing steps may be required by users before further analysis can be performed. Recommended pre-processing steps include: (i) re-orientation of the AutoPi accelerometer axes to align with the principal car axes; (ii) map-matching – where the GPS routes are corrected using the reference measurements; (iii) interpolation – where GPS coordinates are assigned to all sensor readings; and (iv) structuring of data – where sensor readings are re-sampled to ensure consistency between car- and reference data across all sensors.

## 4 Experimental Design, Materials and Methods

**Fig. 4:**
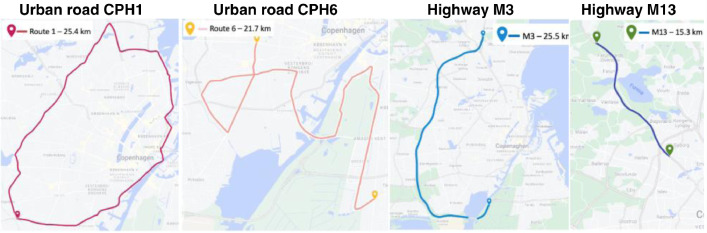
Overview maps of the different routes in LiRA-CD.

The data collection involved capturing car sensor signals while driving and standard road condition parameters for the same roads. The choice of roads was guided by the desire to cover a wide variety of pavement conditions with respect to age, type, and distress severity. The dataset has been used to develop several models to estimate road conditions. In [5], a data-driven method is proposed for estimating the tire-grip potential of asphalt roads utilizing transverse accelerations and VIAFRIK measurements. In [6], a machine-learning model for evaluating pavement friction based on in-vehicle sensor data and images collected with a GoPro camera is proposed. The Authors utilized longitudinal and lateral accelerations, speed, yaw rate, wheel RPM, engine torque, steering angle, and reference measurements from the VIAFRIK vehicle. In [7], vertical accelerations, speed measurements, and GPS location are utilized to estimate the International Roughness Index (IRI). In [8], vertical acceleration readings and a simple dynamic model are utilized for road profile inversion. In both cases, the P79 measurements were used as a reference. In [9] and [10] new road energy efficiency monitoring concepts are proposed based on vehicle speed, longitudinal acceleration, wheel torque, and traction power measurements. Overview maps of the different routes in LiRA-CD are depicted in Fig. 4.

**Fig. 5:**
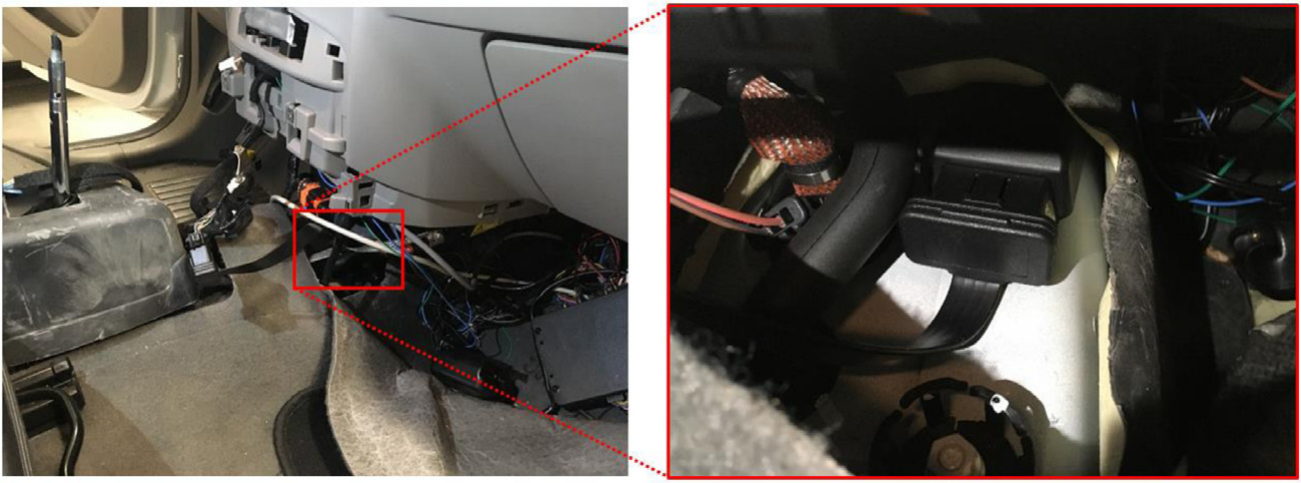
Picture of the passenger side of the Renault Zoe with the access panel to the middle console removed and the location of the AutoPi telematics unit inside the middle console.

In-vehicle sensor data were collected with an AutoPi Telematics Unit (3rd generation) connected to the GM vehicle's controller area network (CAN). This unit includes a single-board Raspberry Pi computer, with added GPS and accelerometer modules - as described in [2]. The AutoPi units were physically fixed to the frame in the middle of the GM car close to the front axle. The installation of the devices is depicted in Fig. 5.

The standard road conditions, i.e., the reference dataset, were collected from vehicles operated by the DRD. The surveying included: (i) P79 Profilometer – a van equipped with a beam hosting 25-point lasers that measure longitudinal and transverse profiles. Two of these lasers operate at an exceptionally high acquisition frequency for measuring surface texture depth. The P79 also measures cross-fall slope, vertical curvature, IRI, mean profile depth, rutting, and offers a 3D view of the entire pavement surface; (ii) ARAN9000 – a multi-functional road scanning vehicle that quantifies road defects and distresses using cameras and a Laser Cracking Measurement System (LCMS); (iii) VIAFRIK – a skid resistance device complying with CEN/TS 15,901–5 standard, and operated with a relative slippage of 20%

The car sensor data were collected during the summer and the autumn of 2020 and the spring and the summer of 2021 utilizing eight different cars. Designated drivers were instructed to drive in the right lane at a constant speed of 90 km/h on the highways and 50 km/h on the urban roads or follow the speed limit (e.g., in case this was lower than the instructed speed) or the traffic (e.g., in the case of congestion). An overview of the road metadata and test instructions during measurement campaigns is shown in Table 7.

**Table 7:** LiRA-CD road metadata and test instructions.

Route	Road length	Road type	Avg. speed [km/h]	Number of cars used	No. of directions	No. of car passes	Total length of road	Driver instruction
CPH1	25.5	Urban	50	7	2	10	510	Keep speed limit or follow traffic, drive in right/heavy lane
CPH6	23.0	Urban and Sub-urban	50	2	2	7	322	Keep speed limit or follow traffic, drive in right/heavy lane
M3	26.0	Highway	90	6	2	10	520	Constant speed of 90 km/h or follow traffic, drive in right/heavy lane
M13	14.5	Highway	90	3	2	10	290	Constant speed of 90 km/h or follow traffic, drive in right/heavy lane
M3	26.0	Highway	60	1	2	1	52	Drive behind VIAFRIK at constant speed of 60 km/h, keep wheels in wet wheel tracks
CPH1	1.5	Urban	50	3	2	39	117	Speed limit or follow traffic, drive in right/heavy lane

An overview of the timing of measurement campaigns for the car data collection, surface and weather conditions are shown in Table 8; the heading ‘TaskId’ refers to a specific test (and could include several passes and directions over the same road section), 'Unit no.' refers to the different AutoPi units (cars) used, and ‘Filename’ refers to the filename in LiRA-CD.

**Table 8:** Overview of data collection with cars.

TaskId	Unit no.	Date	Avg. Air Temp. [°]	Surface	Weather	Filename
5642	TMU#2	03–11–2020	12	Dry	Sunny	M3_HH & M3_VH
5688	TMU#1	08–11–2020	10	Dry	Sunny	M3_HH & M3_VH
7567	TMU#11	16–04–2021	11	Dry	Sunny	M3_HH & M3_VH
7589	TMU#12	17–04–2021	9	Dry	Sunny	M3_HH & M3_VH
7885	TMU#11	24–04–2021	7	Dry	Sunny	M3_HH & M3_VH
7895	TMU#11	24–04–2021	9	Dry	Sunny	M3_HH & M3_VH
7995	TMU#10	26–04–2021	6	Dry	Sunny	M3_HH & M3_VH
8189	TMU#12	30–04–2021	10	Dry	Sunny	M3_HH & M3_VH
8197	TMU#14	30–04–2021	11	Dry	Sunny	M3_HH & M3_VH
8205	TMU#14	30–04–2021	12	Dry	Sunny	M3_HH & M3_VH
6337	TMU#10	15–04–2021	8	Dry	Sunny	M13_HH & M13_VH
7203	TMU#14	15–04–2021	8	Dry	Sunny	M13_HH & M13_VH
7206	TMU#14	15–04–2021	8	Dry	Sunny	M13_HH & M13_VH
7245	TMU#11	15–04–2021	8	Dry	Sunny	M13_HH & M13_VH
7247	TMU#11	15–04–2021	8	Dry	Sunny	M13_HH & M13_VH
8040	TMU#14	27–04–2021	11	Dry	Sunny	CPH1_HH
8227	TMU#11	30–04–2021	9	Dry	Cloudy	CPH1_HH
9289	TMU#6	15–05–2021	9	Dry	Cloudy	CPH1_HH
10,218	TMU#12	20–05–2021	13	Dry	Cloudy	CPH1_HH
10,900	TMU#11	27–05–2021	11	Dry	Cloudy	CPH1_HH
11,360	TMU#7	30–05–2021	13	Dry	Sunny	CPH1_HH
11,367	TMU#7	30–05–2021	16	Dry	Sunny	CPH1_HH
7792	TMU#10	22–04–2021	10	Dry	Sunny	CPH1_VH
8040	TMU#14	27–04–2021	11	Dry	Sunny	CPH1_VH
8049	TMU#14	27–04–2021	11	Dry	Sunny	CPH1_VH
8227	TMU#11	30–04–2021	9	Dry	Cloudy	CPH1_VH
9289	TMU#6	15–05–2021	9	Dry	Cloudy	CPH1_VH
9314	TMU#10	15–05–2021	9	Dry	Cloudy	CPH1_VH
10,204	TMU#12	20–05–2021	14	Dry	Cloudy	CPH1_VH
10,218	TMU#12	20–05–2021	12	Dry	Cloudy	CPH1_VH
13,175	TMU#1	27–08–2021	16	Dry	Sunny	CPH6_HH
13,177	TMU#1	25–08–2021	15	Dry	Cloudy	CPH6_HH
13,178	TMU#7	17–08–2021	17	Dry	Sunny	CPH6_HH
13,179	TMU#1	11–08–2021	19	Dry	Sunny	CPH6_HH
13,180	TMU#1	11–08–2021	18	Dry	Sunny	CPH6_HH
13,196	TMU#1	11–08–2021	17	Dry	Sunny	CPH6_VH
13,197	TMU#1	11–08–2021	16	Dry	Sunny	CPH6_VH
13,198	TMU#7	17–08–2021	16	Dry	Sunny	CPH6_VH
13,199	TMU#7	18–08–2021	15	Dry	Cloudy	CPH6_VH
13,200	TMU#1	25–08–2021	15	Dry	Cloudy	CPH6_VH
13,201	TMU#7	27–08–2021	15	Dry	Sunny	CPH6_VH
7505	TMU#2	15–04–2021	8	Wet	Sunny	task_7505_sensorname
16,006	-	30–08–2022	17	Dry	Sunny	platoon_CPH1_HH & platoon_CPH1_VH
16,008	-	30–08–2022	17	Dry	Sunny	platoon_CPH1_HH & platoon_CPH1_VH
16,009	-	30–08–2022	17	Dry	Sunny	platoon_CPH1_HH & platoon_CPH1_VH
16,010	-	30–08–2022	17	Dry	Sunny	platoon_CPH1_HH & platoon_CPH1_VH
16,011	-	30–08–2022	17	Dry	Sunny	platoon_CPH1_HH & platoon_CPH1_VH

An overview of the timing of measurement campaigns for the reference data, surface and weather conditions are shown in Table 9; the header ‘Filename’ refers to the filename in LiRA-CD.

**Table 9:** Overview of data collection of standard road conditions.

Vehicle	Spatial resolution [m]	Date	Avg. Air Temp. [°]	Surface	Weather	Filename
ARAN9000	1.0	12–08–2020	22	Dry	Sunny	cph1_aran_vh
ARAN9000	1.0	12–08–2020	22	Dry	Sunny	cph1_aran_hh
VIAFRIK	5.0	19–11–2019	6	Dry	Cloudy	cph1_fric_vh
VIAFRIK	5.0	19–11–2019	6	Dry	Cloudy	cph1_fric_hh
P79	10.0	10–09–2020	15	Dry	Sunny	cph1_iri_mpd_rut_vh
P79	10.0	10–09–2020	15	Dry	Sunny	cph1_iri_mpd_rut_hh
P79	0.1	10–09–2020	15	Dry	Sunny	cph1_zp_vh
P79	0.1	10–09–2020	15	Dry	Sunny	cph1_zp_hh
ARAN9000	10.0	30–08–2021	19	Dry	Sunny	cph6_aran_vh
ARAN9000	10.0	30–08–2021	19	Dry	Sunny	cph6_aran_hh
VIAFRIK	5.0	19–11–2019	6	Dry	Cloudy	cph6_fric_vh
VIAFRIK	5.0	19–11–2019	6	Dry	Cloudy	cph6_fric_hh
P79	10.0	04–06–2020	16	Dry	Sunny	cph6_iri_mpd_rut_vh
P79	10.0	04–06–2020	16	Dry	Sunny	cph6_iri_mpd_rut_hh
P79	0.1	04–06–2020	16	Dry	Sunny	cph6_zp_vh
P79	0.1	04–06–2020	16	Dry	Sunny	cph6_zp_hh
ARAN9000	1.0	13–08–2020	23	Dry	Sunny	m3_aran_vh
ARAN9000	1.0	13–08–2020	23	Dry	Sunny	m3_aran_hh
VIAFRIK	5.0	18–11–2019	9	Dry	Cloudy	m3_fric_vh
VIAFRIK	5.0	18–11–2019	9	Dry	Cloudy	m3_fric_hh
P79	10.0	10–09–2020	16	Dry	Sunny	m3_iri_mpd_rut_vh
P79	10.0	10–09–2020	16	Dry	Sunny	m3_iri_mpd_rut_hh
P79	0.1	10–09–2020	16	Dry	Sunny	m3_zp_vh
P79	0.1	10–09–2020	16	Dry	Sunny	m3_zp_hh
ARAN9000	1.0	13–08–2020	23	Dry	Sunny	m13_aran_vh
ARAN9000	1.0	13–08–2020	23	Dry	Sunny	m13_aran_hh
VIAFRIK	5.0	14–08–2020	23	Dry	Sunny	m13_fric_vh
VIAFRIK	5.0	14–08–2020	23	Dry	Sunny	m13_fric_hh
P79	10.0	10–09–2020	16	Dry	Sunny	m13_iri_mpd_rut_vh
P79	10.0	10–09–2020	16	Dry	Sunny	m13_iri_mpd_rut_hh
P79	0.1	10–09–2020	16	Dry	Sunny	m13_zp_vh
P79	0.1	10–09–2020	16	Dry	Sunny	m13_zp_hh
VIAFRIK	5.0	15–04–2021	8	Wet	Sunny	m3_custom_fric_vh
VIAFRIK	5.0	15–04–2021	8	Wet	Sunny	m3_curstom_fric_hh

## Ethics Statement

This work did not include work involved with human subjects, animal experiments or data collected from social media platforms.

## CRediT authorship contribution statement

**Asmus Skar:** Conceptualization, Methodology, Software, Data curation, Supervision, Writing – original draft, Visualization. **Anders M. Vestergaard:** Software, Data curation. **Thea Brüsch:** Software, Data curation. **Shahrzad Pour:** Software, Data curation, Resources, Writing – review & editing. **Ekkart Kindler:** Conceptualization, Supervision. **Tommy Sonne Alstrøm:** Conceptualization, Software, Supervision. **Uwe Schlotz:** Data curation. **Jakob Elsborg Larsen:** Resources, Writing – review & editing. **Matteo Pettinari:** Conceptualization, Methodology, Project administration, Funding acquisition.

## Data Availability

Live Road Assessment Custom Dataset (LiRA-CD) (Original data) (https://data.dtu.dk/collections/Live_Road_Assessment_Custom_Dataset_LiRA-CD_/6659909).
